# Study on the Deposition Uniformity of Triple-Target Magnetron Co-Sputtering System: Numerical Simulation and Experiment

**DOI:** 10.3390/ma15217770

**Published:** 2022-11-04

**Authors:** Guo Zhu, Baijun Xiao, Ganxin Chen, Zhiyin Gan

**Affiliations:** 1 School of Mechanical & Electrical Engineering, Hunan City University, Yiyang 413000, China; 2 School of Mechanical Science & Engineering, Huazhong University of Science & Technology, Wuhan 430074, China

**Keywords:** film thickness uniformity, multi-targets magnetron co-sputtering system, target-substrate distance, target-substrate angle, numerical simulation

## Abstract

The uniformity of magnetron-sputtered films can be evaluated using an analytical model whose key parameters, such as included angle cosine and distance between infinitesimal elements, are so far calculated based on targets-substrate geometric relation. This existing computation scheme is not applicable in a triple-target magnetron co-sputtering system with complex targets-substrate geometric relation. In this work, a computation method was proposed to calculate the deposition uniformity of a triple-target magnetron co-sputtering system based on the analytical model. In this method, the coordinates of the infinitesimal elements on the substrate and targets were calibrated in an identical global coordinate system via coordinate transformation, such that the key parameters of the analytical formula can be evaluated by vector computation. The effects of the target-substrate angle and target-substrate distance on the deposition uniformity of a given triple-target magnetron co-sputtering system were investigated via numerical simulation and experiment, respectively. Simulation results were consistent with experimental results. Relevant evolution mechanisms of the deposition uniformity of the co-sputtering system with the variations of target-substrate parameters were discussed in detail based on the simulation results. It is expected that this computation approach can be employed to provide theoretical guidance for the fast and economical fabrication of high-quality, large-area film and composite films.

## 1. Introduction

Due to its high deposition rate and low manufacturing cost, magnetron sputtering has been widely employed to fabricate optical and electrical film devices [1,2]. The performance of sputtered film devices is significantly influenced by film uniformity [3], which is mainly determined by the parameters of target-substrate configuration [4]. Computer simulation as an alternative approach for conventional experiments can predict the optimal target-substrate configuration of a magnetron sputtering system and thus improve the efficiency of manufacturing high-quality film.

In the past decades, relevant numerical models have been successively established to evaluate the deposition uniformity of single-target magnetron sputtering systems, possessing parallel co-axial target-substrate configuration [5,6,7], parallel off-axis target-substrate configuration [8], angular offset target-substrate configuration [9], rotated substrate [10], or stationary convex substrate [11]. These numerical models assumed that the target material in the erosion groove was uniformly etched. Vasilev et al. [12] tried to optimize the existing numerical model by using a one-time measured topography of the etched target to characterize the sputtering rate distribution of the target material. In reality, the sputtering rate distribution of the target material is proportional to the depth difference between the profiles of the erosion groove before and after each sputtering process, which tends to be small and difficult to accurately measure. Cheng et al. [13] developed a simulation model by postulating that the sputtering rate distribution of the target material was proportional to the horizontal magnetic field intensity on the target surface. However, the non-uniform erosion of the target surface is determined by both the horizontal and vertical magnetic field intensities [14,15,16,17,18]. 

On the other hand, the multiple-targets magnetron co-sputtering system has been extensively used to fabricate high-quality, large-area film [19] and composite films [20]. However, relevant numerical research on the deposition uniformity of the multi-targets magnetron co-sputtering system has rarely been reported. To our knowledge, Zhang et al. [21] proposed a computation scheme for the deposition uniformity of a twin-target co-sputtering system through complex and rigid mathematical deduction based on target-substrate geometric relation. This computation scheme can hardly be extended to the triple-target co-sputtering system due to its more complicated target-substrate configuration. In this context, this work proposed a computation method to calculate the deposition uniformity of the triple-target magnetron co-sputtering system. In this method, vector computation was utilized to calculate the key parameters of the analytical model, and the sputtering rate distribution of the target material was characterized reasonably. This computation scheme was employed to investigate the evolution mechanisms of the deposition uniformity of a given triple-target magnetron co-sputtering system with the changes of target-substrate angle and target-substrate distance. This work is expected to provide theoretical guidance on the fast fabrication of high-quality, large-area film and composite films by a triple-target magnetron co-sputtering system.

## 2. Model and Method

### 2.1. Analytical Model

The numerical model to calculate the film thickness distribution will be derived based on the following assumptions:

1. The sputtering rate distribution of the target material, which is dependent on both the horizontal and vertical magnetic field intensities on the target surface, can be described as below [22]:
  (1) $$P(x_{t},y_{t})\propto \frac{B_{r}(x_{t},y_{t})}{B_{r}^{2}\left(x_{t},y_{t}\right)+B_{z}^{2}\left(x_{t},y_{t}\right)}$$
  where *B_r_*(*x_t_,y_t_*) and *B_z_*(*x_t_,y_t_*) represent the horizontal and vertical magnetic field intensities on the target surface, respectively.
2. The emission angles of the sputtered particles from the target surface obey the modified angular distribution proposed by Yamamura [23]:
  (2) $$S(\theta )=\cos\theta (1+\xi \cos^{2}\theta )$$
  where *θ* denotes the emission polar angle of a sputtered particle, and *ξ* is a fitting parameter as a function of bombarding energy [23].
3. The deposition rate of sputtered particles onto a substrate surface has a negative exponent relationship with path length *L* [24]:
  (3) $$\varphi (L)\propto \exp(-L/\lambda_{m})$$
  (4) $$\lambda =\frac{k_{B}T_{g}}{P\sigma }$$
  where *λ*_m_ is the mean free path of the sputtered particle, *k_B_* is the Boltzmann constant, *σ* is the cross-section for momentum transfer, and *T_g_* and *P* are the temperature and pressure of the background gas, respectively.
4. Sputtered particles are immediately condensed when they arrive at the substrate.

**Figure 1:**
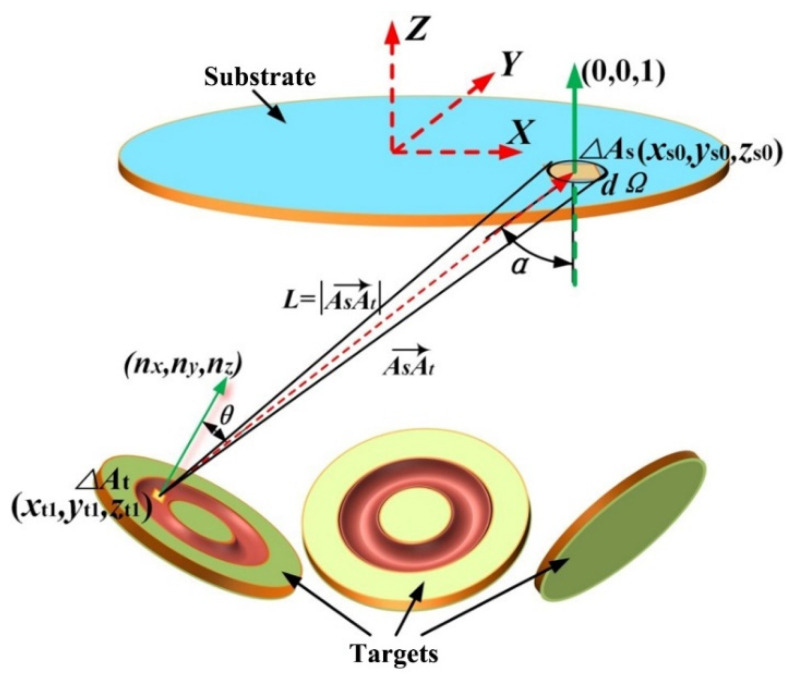
Geometric structure of triple-target magnetron co-sputtering system.

Figure 1 displays the geometric structure of the triple-target magnetron co-sputtering system used in the present numerical simulation. According to assumptions 1–3 and the geometric relation as shown in Figure 1, the flux density of the sputtered particles, which are ejected from an infinitesimal element ΔA_t_ of the target surface and ultimately deposited onto an infinitesimal element ΔA_s_ of the substrate surface, can be given as below:

(5) $$d\varphi ={MP(x}_{t},y_{t})S(\theta )\exp({-L/\lambda }_{m}{)\mathrm{dA}}_{t}d\Omega /2\pi$$

(6) $$M=\bar{J_{i}}YA_{t}m_{t}/e$$

(7) d*Ω*= dA_s_cos*α*/*L*^2^

where *M* is the mass of the sputtered particles ejected from the target surface in unit time, $\bar{J_{i}}$ is the mean ion current density, *Y* is the sputtering yield of the target material, A_t_ is the area of the target surface, *m*_t_ is the mass of the target atom, *e* is the charge of an electron, *Ω* is a solid angle, and *α* is the angle of vector A_s_A_t_ with respect to the normal of the substrate surface. According to Equation (6), d*φ* represents the mass of the sputtered particles deposited onto an infinitesimal element ΔA_s_ of the substrate surface in unit time.

Substituting Equations (2) and (7) into Equation (5), d*φ* can be further expressed as:

(8) $$d\varphi =\frac{MP(x_{t},y_{t})\cos\theta (1+\varepsilon \cos^{2}\theta )\cos\alpha \exp({-L/\lambda }_{m}{)\mathrm{dA}}_{t}{\mathrm{dA}}_{s}}{2\pi L^{2}}$$

(9) $$L=\sqrt{{\left(x_{s0}-x_{t1}\right)}^{2}+{\left(y_{s0}-y_{t1}\right)}^{2}+{\left(z_{s0}-z_{t1}\right)}^{2}}$$

(10) $$\cos\theta =\frac{\vec{A_{s}A_{t}}\cdot {\vec{n}}_{t}}{\left|\vec{A_{s}A_{t}}\right|}=\frac{(x_{s0}-x_{t1})n_{\mathrm{tx}}+(y_{s0}-y_{t1})n_{ty}+(z_{s0}-z_{t1})n_{\mathrm{tz}}}{\sqrt{{\left(x_{s0}-x_{t1}\right)}^{2}+{\left(y_{s0}-y_{t1}\right)}^{2}+{\left(z_{s0}-z_{t1}\right)}^{2}}}$$

(11) $$\cos\alpha =\frac{\vec{A_{s}A_{t}}\cdot {\vec{n}}_{s}}{\left|\vec{A_{s}A_{t}}\right|}=\frac{\left|z_{s0}-z_{t}\right|}{\sqrt{{\left(x_{s0}-x_{t1}\right)}^{2}+{\left(y_{s0}-y_{t1}\right)}^{2}+{\left(z_{s0}-z_{t1}\right)}^{2}}}$$

According to assumption 4 and the conservation of the particle number, the flux density of the sputtered particles, which are ejected from an infinitesimal element ΔA_t_ of the target surface and ultimately deposited onto the infinitesimal element ΔA_s_ of the substrate surface, can also be expressed as:

(12) d*φ* = *ρ*d*T*dA_s_

where *ρ* is the density of the deposited film and d*T* is the thickness of the film deposited onto the infinitesimal element ΔA_s_.

Then, the film thickness within the infinitesimal element ΔA_s_, due to the deposition of the sputtered particles from the infinitesimal element ΔA_t_, can be obtained based on Equations (8) and (12):

(13) $$dT=\frac{MP(x_{t},y_{t})\cos\theta (1+\varepsilon \cos^{2}\theta )\cos\alpha \exp({-L/\lambda }_{m}{)\mathrm{dA}}_{t}}{2\pi \rho L^{2}}$$

Accordingly, due to the deposition of the sputtered particles from three target surfaces, the total film thickness within the infinitesimal element ΔAs (*x*_s0_, *y*_s0_) can be expressed as:

(14) $$T_{T}(x_{s0},y_{s0})=\iint_{S_{\mathrm{At}}+S_{\mathrm{Bt}}+S_{\mathrm{Ct}}}\frac{MP{(x}_{t},y_{t})\cos\theta (1+\varepsilon \cos^{2}\theta )\cos\alpha \exp(-L/\lambda_{m})}{2\pi \rho L^{2}}{\mathrm{dA}}_{t}$$

where S_At_, S_Bt_, and S_Ct_ represent the integral domains on the three targets, respectively. As the substrate rotates around its axis, the total film thickness within the infinitesimal element ΔAs (x_s0_ = *r*_s0_cos*ω*_s0_,y_s0_ = *r*_s0_sin*ω*_s0_) can be written as [20]:

(15) $$T(r_{s0},\omega_{s0})=\frac{1}{2\pi }\int_{0}^{2\pi }T_{T}(r_{s0},\omega_{s})d\omega_{s}$$

where *r*_s0_ is the radial distance between the infinitesimal element ΔA_s_ and the substrate center, and *ω*_s0_ is the azimuth angle of the infinitesimal element ΔA_s_.

Therefore, as long as the coordinate values of the infinitesimal elements on the substrate and three targets in an identical global coordinate system are determined, cos*α* and cos*θ* in Equation (14) can be conveniently calculated by Equations (10) and (11), respectively. Then, the film thickness distribution on the substrate can be further evaluated by Equations (14) and (15).

### 2.2. Global Coordinates of the Infinitesimal Elements on Substrate and Three Targets

**Figure 2:**
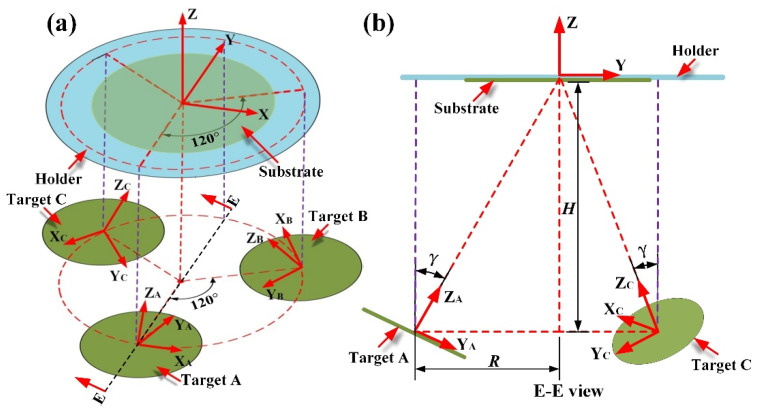
Target-substrate geometric configuration of a triple-target magnetron co-sputtering system: (**a**) Isometric view; (**b**) Projected view through E-E cross section.

Figure 2 displays the target-substrate geometric configuration of the triple-target magnetron co-sputtering system. In Figure 2, a global coordinate system (XYZ) and three local coordinate systems (X_A_Y_A_Z_A_, X_B_Y_B_Z_B_, and X_C_Y_C_Z_C_) are established. In the global coordinate system, origin is located at the substrate center in the middle of the substrate holder, and the Z axis is aligned to the normal of the substrate surface. In each local coordinate system, the origin is located at the center of the target surface, and the Z axis is aligned to the normal of the corresponding target surface. *γ* represents the angle of the Z axis of each local coordinate system, with respect to that of the global coordinate system. 

It is known that the dot product between a vector and a unit vector represents the projection length of the vector onto the unit vector, i.e., the coordinate value of the vector on a coordinate axis. According to the geometric relations as shown in Figure 2, in the local coordinate system X_A_Y_A_Z_A_, the unit vectors along the X, Y, and Z axes of the global coordinate system XYZ can be written as (1, 0, 0), (0, cosγ, sinγ), and (0, −sinγ, cosγ), respectively. If the coordinate of the infinitesimal element ΔA_At_ on the target A in the local coordinate system X_A_Y_A_Z_A_ is marked as (*x*_A0_, *y*_A0_, *z*_A0_), then its coordinate in the global coordinate system XYZ can be given by:

(16) $$\left[\begin{array}{c} x_{A1}\\ y_{A1}\\ z_{A1} \end{array}\right]=\left[\begin{array}{ccc} 1 & 0 & 0\\ 0 & \cos\gamma & \sin\gamma \\ 0 & -\sin\gamma & \cos\gamma \end{array}\right]\left[\begin{array}{c} x_{A0}\\ y_{A0}\\ z_{A0} \end{array}\right]+\left[\begin{array}{c} 0\\ -R\\ -H \end{array}\right]$$

where *R* and *H* denote the horizontal and vertical distances between the centers of the target and substrate, respectively. Similarly, in the global coordinate system XYZ, the coordinates of the infinitesimal elements ΔA_Bt_ and ΔA_Ct_ on the target B and C can be expressed as below:

(17) $$\left[\begin{array}{c} x_{B1}\\ y_{B1}\\ z_{B1} \end{array}\right]=\left[\begin{array}{ccc} -\sin30{}^{\circ} & -\cos30{}^{\circ}\cos\gamma & -\cos30{}^{\circ}\sin\gamma \\ \cos30{}^{\circ} & -\sin30{}^{\circ}\cos\gamma & -\sin30{}^{\circ}\sin\gamma \\ 0 & -\sin\gamma & \cos\gamma \end{array}\right]\left[\begin{array}{c} x_{B0}\\ y_{B0}\\ z_{B0} \end{array}\right]+\left[\begin{array}{c} R\cos30{}^{\circ}\\ R\sin30{}^{\circ}\\ -H \end{array}\right]$$

(18) $$\left[\begin{array}{c} x_{C1}\\ y_{C1}\\ z_{C1} \end{array}\right]=\left[\begin{array}{ccc} -\sin30{}^{\circ} & \cos30{}^{\circ}\cos\gamma & \cos30{}^{\circ}\sin\gamma \\ -\cos30{}^{\circ} & -\sin30{}^{\circ}\cos\gamma & -\sin30{}^{\circ}\sin\gamma \\ 0 & -\sin\gamma & \cos\gamma \end{array}\right]\left[\begin{array}{c} x_{C0}\\ y_{C0}\\ z_{C0} \end{array}\right]+\left[\begin{array}{c} -R\cos30{}^{\circ}\\ R\sin30{}^{\circ}\\ -H \end{array}\right]$$

## 3. Experiment

Cu thin films were prepared by a triple-target DC magnetron co-sputtering system with three never-used copper targets (purity of 99.99%, diameter of 60 mm, and thickness of 5 mm) under different target-substrate angles and target-substrate distances. A 101.6 mm p-type single crystal (100) silicon wafer with a thickness of 0.5 mm was used as the substrate. A total of eight experiments were conducted and divided into two groups. In experimental group 1, the target-substrate distance was kept at 70 mm and the target-substrate angles were set to 15°, 25°, 35°, and 45°, respectively. In experimental group 2, the target-substrate angle was kept at 35° and the target-substrate distances were set to 50 mm, 70 mm, 90 mm, and 110 mm, respectively. 

**Figure 3:**
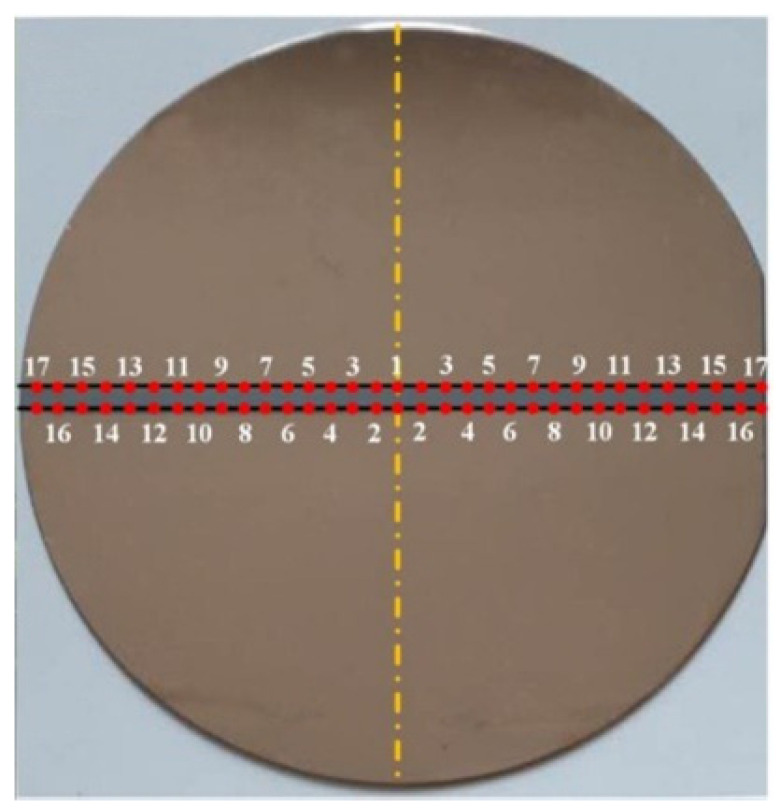
Test points of film thickness on the 2 steps in the deposited Cu film. Yellow dashed line denotes the symmetry axis of substrate.

Prior to sputtering deposition, the silicon substrate was ultrasonically cleaned in acetone for 5 min and then rinsed in de-ionized water. The polyimide film, with a width of 3 mm, was pasted onto the middle region of the substrate surface to form 2 steps in the deposited film, as shown in Figure 3. Then, silicon substrate was placed in the middle of the substrate holder. In each experiment, the base pressure of the vacuum chamber was pumped down to 5 × 10^−4^ Pa using a turbomolecular pump. During the sputtering deposition, the sputtering current, target voltage, and argon gas pressure were kept at 0.26 A, 400 V, and 0.5 Pa, respectively. The film deposition time was set to 10 min. 

The thickness of the sputtered Cu film was quantified by measuring the step height [25,26] on the substrate with the deposited Cu film using a Bruker DektakXT surface profilometer (Billerica, MA, USA). In order to obtain the radial distribution of the film thickness, 33 equally spaced test points were chosen on each step edge. These test points were numbered from 1 to 17 from substrate center to substrate margin. Thus, the 4 test points labeled by an identical number had the same radial distance to the substrate center, and the mean value of the film thickness at the 4 test points was evaluated as the film thickness at that radial distance. 

**Figure 4:**
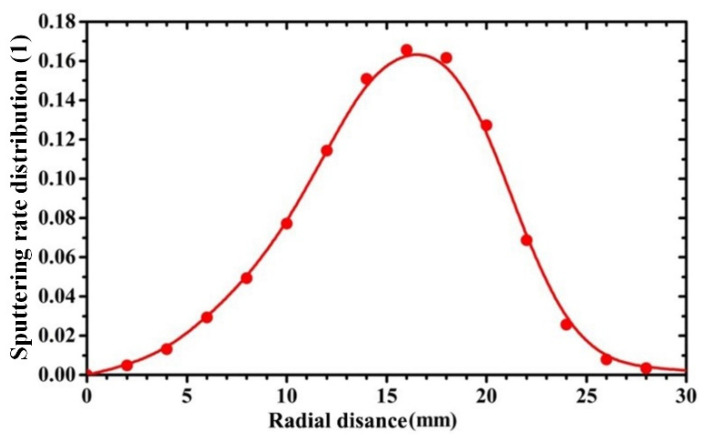
Sputtering rate distribution of the target material along the radial direction of the target surface.

The magnetic field intensities on the target surfaces were measured by a Hall sensor [27] whose margin of error and maximal measuring range is 2% and 2400 mT, respectively. Figure 4 displays the normalized sputtering rate distribution of the target material along the radial direction of the target surface, which was calculated based on Equation (1).

## 4. Results and Discussion

### 4.1. Effect of Target-Substrate Angle on Film Thickness Distribution

**Figure 5:**
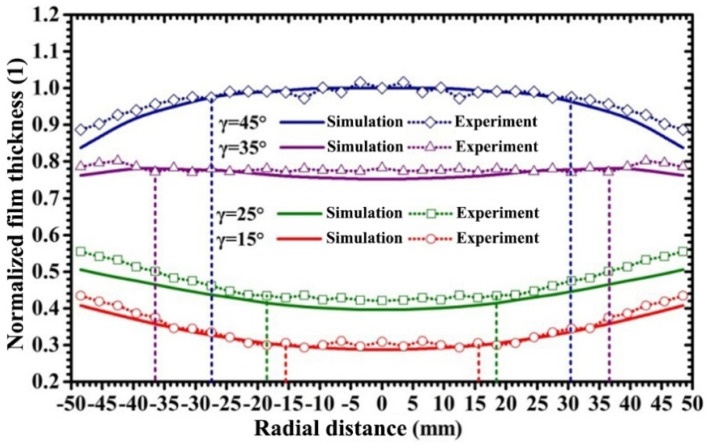
Normalized film thickness distribution curves under various target-substrate angles.

Figure 5 displays the film thickness distribution curves under various target-substrate angles as the target-substrate distance is kept at 70 mm. In Figure 5, solid curves and dotted curves represent the film thickness distribution curves obtained by simulative calculation and experimental measurement, respectively. The calculated film thickness represents the thickness of the film deposited onto the substrate in unit time; thus, in order to compare the simulation and experimental results, the calculated values of film thickness were normalized by that of the film thickness at the substrate center at the target-substrate angle of 45°, and the measured values of the film thickness were normalized by that of the film thickness at the substrate center at the target-substrate angle of 45°. It can be seen that the simulation results are not exactly consistent with the experimental results. In magnetron sputtering, the deposited particles can be classified as fast-moving particles (not collide) and slow-moving particles (collide) [7], and the latter are the scattered particles which are transported by diffusion. The mean free path of sputtered particles is approximately 17 mm at 0.5 Pa and 300 K, which suggests that the diffusion effect of scattered particles (slow-moving particles) may influence the distribution of film thickness. The scattering of sputtered particle flux was not taken into account in our simulation, which may be the reason for the deviation between the calculated and measured thickness values at the edge of the substrate. However, the maximum deviation between the simulation and experimental results was less than 8%, which indicates that the simulation results can sufficiently predict the actual film thickness distribution.

As shown in Figure 5, as the target-substrate angle increases from 15° to 45°, the thickness distribution profile of the sputtered film gradually varies from a U-shaped curve to a saddle-shaped curve, and then to an arch-shaped curve. As the target-substrate angle increases from 15° to 35°, the radius of the circular region with the fluctuation of film thickness less than 3% increases from 15.5 mm to 36.5 mm; as the target-substrate angle increases from 35° to 45°, the radius of the circular region with the fluctuation of film thickness less than 3% decreases from 36.5 mm to 27.5 mm. This indicates that when the target-substrate distance is fixed, film uniformity is improved with the proper increase of the target-substrate angle but reduced as the target-substrate angle exceeds a certain value. Furthermore, it is interesting that the sputtering parameters and deposition time were set identically in all the experiments under the 4 target-substrate angles, but the thickness of the sputtered film (the deposition rate of sputtered particles) increased significantly as the target-substrate angle increased from 15° to 45°.

**Figure 6:**
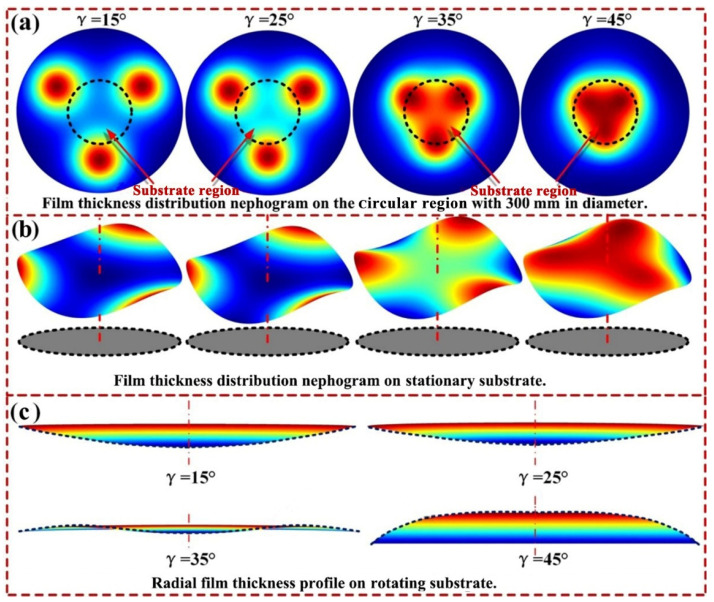
Evolution mechanisms of the deposition uniformity of triple-target magnetron co-sputtering system with target-substrate angle. (**a**) Film thickness distribution nephograms on the circular region with a diameter of 300 mm; (**b**) 3D nephograms of the film thickness distribution on the stationary substrate; (**c**) Section profiles of the film thickness distribution on the rotating substrate.

In order to explore the variation mechanism of film uniformity and deposition rate with a target-substrate angle, the thickness distributions of films deposited on the stationary substrate (substrate does not rotate) under different target-substrate angles were calculated. The thickness distribution nephograms of the films deposited on the circular region with a diameter of 300 mm under different target-substrate angles are displayed in Figure 6a, in which the substrate regions are marked with circular dotted lines. Figure 6b,c, depict the 3D nephograms and corresponding section profiles of the film thickness distribution on the stationary substrate, respectively. It can be seen from the leftmost subgraph of Figure 6a that, when the target-substrate angle is 15°, the film thickness distribution nephogram on the circular region has three 120° uniformly distributed peak points, which are outside the substrate region, and the film thickness values near these peak points are significantly greater than that at the substrate center. This indicates that when the target-substrate angle is 15°, a considerable part of the sputtered particles from three targets are not deposited within the substrate region. Accordingly, the loss of sputtered particles leads to the low deposition rate of sputtered particles on the substrate, and the preferential deposition of sputtered particles near the edge of the substrate results in the U-shaped film thickness distribution on the rotating substrate. As the target-substrate angle increases from 15° to 45°, the three peaks of film thickness distribution nephogram gradually enter the substrate region and approach the substrate center. This allows more sputtered particles to be deposited on the substrate, resulting in a significant increase of the deposition rate on the substrate. Furthermore, as the three peak points of the film thickness distribution nephogram gradually approach the substrate center, the sputtered particle flows from the three sputtering targets superimpose in the region near the substrate center, leading to the significant increase of the film thickness at this region. Consequently, the film thickness profile on the rotating substrate gradually changes from a U-shaped curve to a saddle-shaped curve, and then to an arch-shaped curve, as shown in Figure 6c.

### 4.2. Effect of Target-Substrate Distance on Film Thickness Distribution

**Figure 7:**
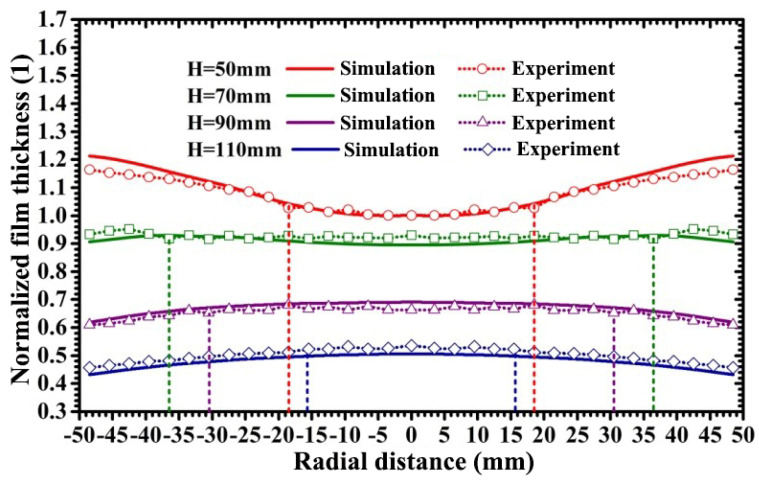
Normalized film thickness distribution curves under various target-substrate distances.

Figure 7 displays the film thickness distribution curves under various target-substrate distances as the target-substrate angle is kept at 35°. In Figure 7, solid curves and dotted curves represent the film thickness distribution curves obtained by the simulative calculation and experimental measurement, respectively. The calculated film thickness represents the thickness of the film deposited onto the substrate in unit time; thus, in order to compare the simulation and experimental results, the calculated values of film thickness were normalized by that of the film thickness at the substrate center at the target-substrate distance of 50 mm, and the measured values of film thickness were normalized by that of the film thickness at the substrate center at the target-substrate distance of 50 mm. It can be seen that the simulation results mostly agreed with the experimental results, and that the maximum deviation between the simulation and experimental results was less than 6%. As shown in Figure 7, as the target-substrate distance increases from 50 mm to 110 mm, the thickness distribution profile of the sputtered films gradually varies from a U-shaped curve to a saddle-shaped curve, and then to an arch-shaped curve. As the target-substrate distance increases from 50 mm to 70 mm, the radius of the circular region with the fluctuation of film thickness less than 3% increases from 18.5 mm to 36.5 mm; as the target-substrate distance increases from 70 mm to 110 mm, the radius of the circular region with the fluctuation of film thickness less than 3% decreases from 36.5 mm to 15.5 mm. This indicates that when the target-substrate angle is fixed, film uniformity is improved with the proper increase of the target-substrate distance but reduced as the target-substrate distance exceeds a certain value. In addition, the increase of the target-substrate distance from 50 mm to 110 mm increases the possibility of the scattering collision of sputtered atoms with background gas atoms, which is responsible for the decrease in the deposition rate of sputtered particles.

**Figure 8:**
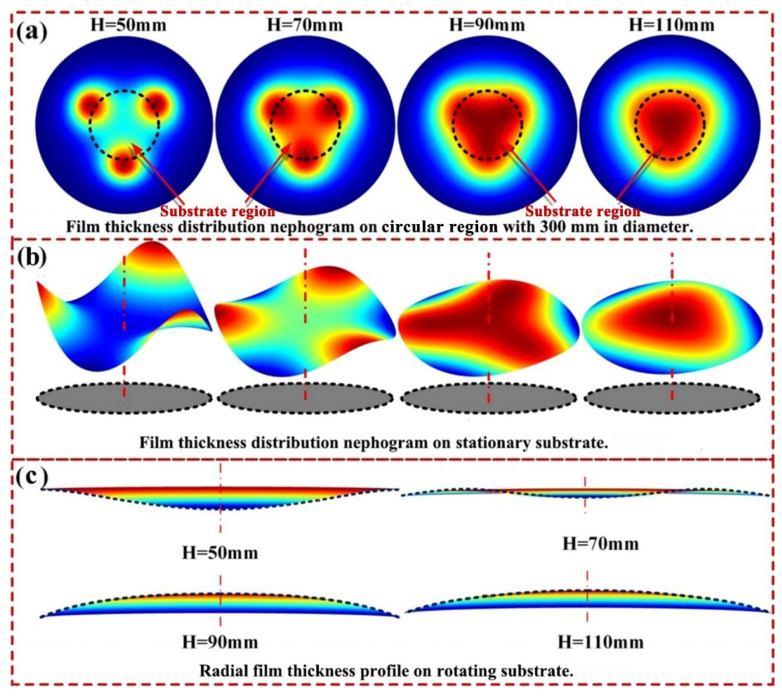
Evolution mechanisms of the deposition uniformity of the triple-target magnetron co-sputtering system with the target-substrate distance. (**a**) Film thickness distribution nephograms on the circular region with a diameter of 300 mm; (**b**) 3D nephograms of the film thickness distribution on the stationary substrate; (**c**) Section profiles of the film thickness distribution on the rotating substrate.

In order to explore the variation mechanism of film uniformity and deposition rate with target-substrate distance, the thickness distributions of films deposited on the stationary substrate (substrate does not rotate) under different target-substrate distances were calculated. The thickness distribution nephograms of the films deposited on the circular region with a diameter of 300 mm under different target-substrate distances are displayed in Figure 8a, in which substrate regions are marked with circular dotted lines. Figure 8b,c, depict the 3D nephograms and the corresponding section profiles of the film thickness distribution on the stationary substrate, respectively. It can be seen from the leftmost subgraph of Figure 8a that, when the target-substrate distance is 50 mm, the film thickness distribution nephogram on the circular region has three 120° uniformly distributed peak points, which are near the margin of the substrate, and the film thickness at these peak points are significantly greater than that at the substrate center. This indicates that when the target-substrate distance is 50 mm, a considerable part of the sputtered particles from three targets are not deposited within the substrate region. Accordingly, the loss of sputtered particles leads to the low deposition rate of sputtered particles on the substrate, and the preferential deposition of sputtered particles near the edge of the substrate results in the U-shaped film thickness distribution on the rotating substrate. As target-substrate distance increases from 50 mm to 110 mm, the three peaks of film thickness distribution nephograms gradually enter the substrate region and approach the substrate center. This allows more sputtered particles to be deposited on the substrate, resulting in a significant increase of the deposition rate on the substrate. Furthermore, as the three peak points of film thickness distribution nephograms gradually approach the substrate center, the sputtered particle flows from the three sputtering targets superimpose in the region near the substrate center, leading to the significant increase of the film thickness at this region. Consequently, the film thickness profile on the rotating substrate gradually changes from a U-shaped curve to a saddle-shaped curve, and then to an arch-shaped curve, as shown in Figure 8c.

## 5. Discussion

**Figure 9:**
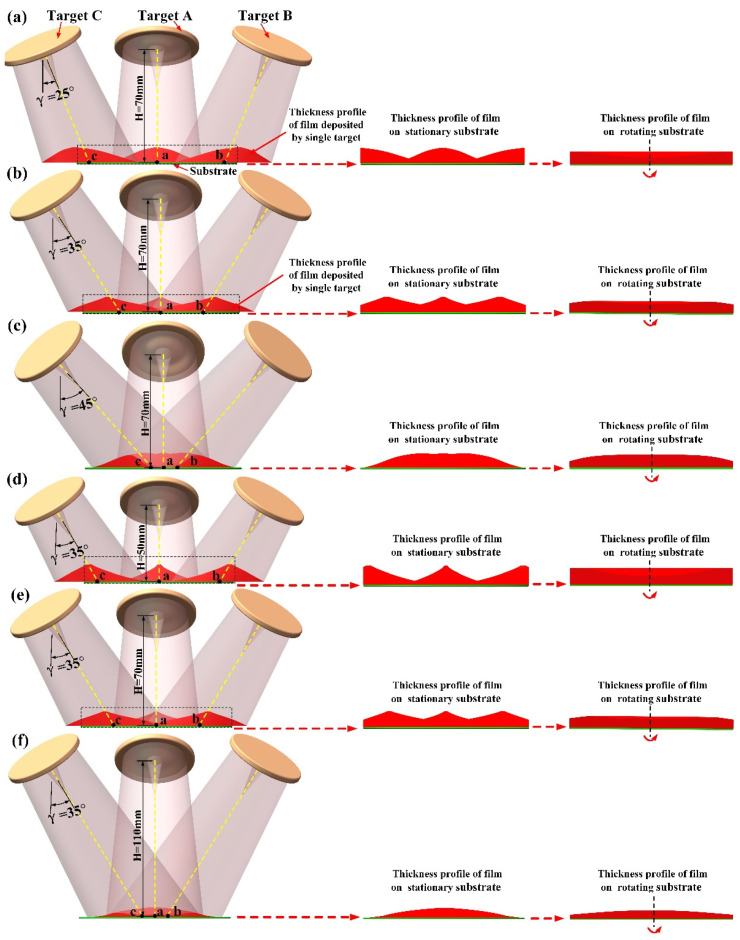
Schematic diagram for the formation mechanism of film thickness distribution: (**a**) H = 70 mm and *γ* = 25°; (**b**) H = 70 mm and *γ* = 35°; (**c**) H = 70 mm and *γ* = 45°, (**d**) *γ* = 35° and H = 50 mm; (**e**) *γ* = 35° and H = 70 mm; (**f**) *γ* = 35° and H = 110 mm.

Figure 9 displays the schematic diagram to further explain the influence-mechanism of the target-substrate angle and target-substrate distance on the uniformity of deposition film. In each subgraph of Figure 9, *γ* denotes target-substrate angle, H denotes target-substrate distance, the dashed yellow lines denote the central axes of targets, the solid green line denotes the substrate, and the three red mound-shaped graphs represent the film thickness profiles of the films deposited on the stationary substrate by the three targets, respectively; points ‘a’, ‘b’, and ‘c’ are the intersection points between the central axes of the three targets and the substrate surface. For the case of the sputtering of a single 50.4 mm target, the maximum flux density of sputtered particles appears at the overlap region of the sputtered particle flow near the central axis of the target [28], and thus the maximum thickness of the deposited film appears at the intersection point between the central axis of the target and the substrate surface [29]. As shown in Figure 9a–c, when the target-substrate distance is kept at 70 mm, with the increase of the target-substrate angle, points ‘a’, ‘b’, and ‘c’ gradually approach the substrate center. As shown in Figure 9d–f, when the target-substrate angle is kept at 35°, with the increase of the target-substrate distance, points ‘a’, ‘b’, and ‘c’ gradually move toward the substrate center. Accordingly, the influence-mechanism of the target-substrate angle on the film thickness distribution, under a constant target-substrate distance, resembles that of the target-substrate distance as the target-substrate angle is kept at a constant. This reveals the reason why the three peak points on the film thickness distribution nephograms in Figure 6a and Figure 8a gradually move toward the substrate center with the increase of the target-substrate angle and target-substrate distance, respectively. In Figure 9a,d, the target-substrate angle and distance are set to (25°, 70 mm) and (35°, 50 mm), respectively. The sputtered particles flow deposited on the edge of the stationary substrate is significantly greater than that deposited at the center of the stationary substrate. Consequently, deposited film presents a U-shaped thickness profile when the substrate revolves around its central axis. When the target-substrate angle gradually increases from 25° to 45° (as shown in Figure 9a–c) and the target-substrate distance gradually increases from 50 mm to 100 mm (as shown in Figure 9d–f), since the points ‘a’, ‘b’, and ‘c’ gradually move towards the substrate center, the sputtered particle flow deposited near the center of the stationary substrate gradually equals and ultimately exceeds that deposited on the edge of the stationary substrate. Therefore, the thickness profile of the deposited film on the rotating substrate varies from a saddle-shaped curve to an arch-shaped curve. 

## 6. Conclusions

In this work, a computation method was proposed to calculate the deposition uniformity of the triple-target magnetron co-sputtering system based on the analytical model. In this method, coordinate transformation was used to determine the coordinates of the infinitesimal elements on the surfaces of a substrate and three targets in an identical global coordinate system. Then, the included angle cosines in the analytical model were evaluated by the cosine formula of the included angle between two vectors, while the distance between the infinitesimal elements were calculated by a computation formula of vector magnitude. In this way, the deposition uniformity of the triple-target magnetron co-sputtering system can be conveniently calculated without complicated geometric derivation. The effects of target-substrate angle and target-substrate distance on the deposition uniformity of a given triple-target magnetron co-sputtering system were investigated via numerical simulation and experiment, respectively. Simulation results were mostly consistent with experimental results. As the target-substrate distance was kept at 70 mm and the target-substrate angle increased from 15° to 45°, the deposition uniformity of the co-sputtering system increased first and then decreased. Furthermore, as the target-substrate angle was kept at 35° and the target-substrate distance increased from 50 mm to 110 mm, the deposition uniformity of the co-sputtering system increased first and then decreased. The optimized uniformity of the sputtered film can be obtained as the target-substrate angle and target-substrate distance are set to 35° and 70 mm, respectively. Simulation results suggest that, with the increase of the target-substrate angle and target-substrate distance, the three peak points of sputtering particle flow from the three sputtering targets gradually approach the substrate center, resulting in the gradual transformation of the film thickness profile from a U-shaped curve to an arch-shaped curve. It is expected that this computation approach can be employed to provide theoretical guidance on the fast fabrication of high-quality, large-area film and composite films by a triple-target magnetron co-sputtering system.

## Data Availability

Not applicable.
